# Retraction Note: Electrochemical Sensor for Detection of miRs Based on the Differential Effect of Competitive Structures in The p19 Function

**DOI:** 10.1038/s41598-021-83097-0

**Published:** 2021-02-16

**Authors:** E. Ghazizadeh, R. K. Oskuee, M. R. Jaafari, S. Hosseinkhani

**Affiliations:** 1 Department of Medical Biotechnology, School of Medicine, Mashhad University of Medical Sciences, Mashhad, Iran; 2 Biotechnology Research Center, Pharmaceutical Technology Institute, Mashhad University of Medical Sciences, Mashhad, Iran; 3 Department of Pharmaceutical Nanotechnology, School of Pharmacy, Mashhad University of Medical Sciences, Mashhad, Iran; 4 Department of Biochemistry, Faculty of Biological Sciences, Tarbiat Modares University, Tehran, Iran

Retraction of: *Scientific Reports* 10.1038/s41598-018-22098-y, published online 28 February 2018

The Editors have retracted this article.

Concerns were raised regarding a number of figures, specifically:

- Figure 2C appears to show repeated features;
- Figure 2E appears to show repeated features;
- Figure 2F appears to show repeated features;
- Figure 3B appears to show an unexpected irregularity in the top right corner.

Additionally, the article shows significant overlap with an article that was simultaneously under consideration with another journal^1^. The Editors therefore no longer have confidence in the reliability of the data reported in the article.

Elham Ghazizadeh disagrees with the retraction. The other authors did not respond to the correspondence about the retraction.
